# A wearable sensor and framework for accurate remote monitoring of human motion

**DOI:** 10.1038/s44172-024-00168-6

**Published:** 2024-01-30

**Authors:** Maximilian Gießler, Julian Werth, Bernd Waltersberger, Kiros Karamanidis

**Affiliations:** 1 Department of Mechanical and Process Engineering, Offenburg University of Applied Sciences, Offenburg, Germany; 2 Sport and Exercise Science Research Centre, School of Applied Sciences, London South Bank University, London, UK; 3 Department of Sport Science, Faculty of Mathematics and Natural Sciences, University of Koblenz, Koblenz, Germany

**Keywords:** Mechanical engineering, Biomedical engineering, Mathematics and computing

## Abstract

Remote monitoring and evaluation of human motion during daily life require accurate extraction of kinematic quantities of body segments. Current approaches use inertial sensors that require numerical time differentiation to access the angular acceleration vector, a mathematical operation that greatly increases noise in the acceleration value. Here we introduce a wearable sensor that utilises a spatially defined cluster of inertial measurement units on a rigid base for directly measuring the angular acceleration vector. For this reason, we used computational modelling and experimental data to demonstrate that our new sensor configuration improves the accuracy of tracking angular acceleration vectors. We confirmed the feasibility of tracking human movement by automatic assessment of experimental fall initiation and balance recovery responses. The sensor therefore presents an opportunity to pioneer reliable assessment of human movement and balance in daily life.

## Abstract (web summary)

Maximilian Gießler and colleagues introduce a wearable sensor to track human movement by directly measuring the angular acceleration. This approach eliminates the noise associated with numerical differentiation. The authors show that the improved tracking accuracy leads to benefits when assessing balance recovery responses to perturbed locomotion.

## Introduction

Human motion in daily life usually consists of a combination of arbitrary translational and rotational movements of various body segments^1^. Given that we abstract the segments of a human body as rigid bodies we can use mechanics to evaluate segment dynamics. This type of evaluation requires quantification of angular velocity, linear and, angular acceleration states of several body segments. Optical motion capture systems have been widely used in analysing body segment kinematics. This type of measurement is the gold standard for direct measurement of position vectors and segment orientation is thence derivable. However, for this approach, several weaknesses arise in other applications. On the one hand, we can assess the velocity or acceleration states of body segments indirectly using the technique of numerical differentiation^1^. In general, it is known that numerical differentiation is noise-amplifying and lowers signal-to-noise ratio. This applies even more so for the required second numerical derivative to evaluate acceleration signals. On the other hand, a motion capture system is location-bounded and can only cover a limited measurement space, such as in a laboratory or clinical environment.

To assess kinematic quantities of body segments during movement in everyday life and outside the laboratory, wearable sensors in the form of inertial measurement units (IMU) are typically used^2,3^. Such IMUs have been widely applied to analyse upper and lower extremity joint kinematics during movement using various population groups and with various research objectives^4–7^.

Several assumptions and limitations must be considered for the use of IMUs to assess kinematic quantities. An IMU includes at least one three-dimensional accelerometer to measure the linear acceleration vector and one gyroscope to measure the angular velocity vector of the body segment to which it is attached. To calculate the angular acceleration vector from the measured values of a single IMU, a numerical time derivative is inevitable. Here it also applies that the numerical differentiation amplifies the noise behaviour of the physical sensor signals.

Numerical differentiation has another disadvantage. The calculation depends on at least two discrete points in time. If backward differentiation is used for real-time application, this will lead to a phase delay in the target signal and the noise amplification effect requires filtering of the signal. If real-time evaluation is required for a person during testing, the phase delay will further increase depending on the filtering principle used. For determining the segment orientation based on the measured signals common approaches use a sensor fusion algorithm. These approaches involve numerical integration (solving a differential equation system for quaternions) using the directly measured angular velocity and fusing it with the sensor orientation estimation derived from evaluating the gravity vector and the earth’s magnetic field. Moreover, the numerical integration of noisy sensor data leads to a signal drift.

In robotics, there are clear examples showing the negative influence of typical white noise behaviour of inertial sensors in combination with numerical differentiation^8^. For example, in robotics numerical differentiation of inertial sensor signals is necessary for calculating the inverse dynamics, e.g. to estimate the centre of pressure or the ground reaction force vector to derive stability criteria. Besides, these calculations must be feasible online in robotics and preferably should not introduce a phase delay. Finally, the noise-amplifying effect makes the estimation of these indicators almost impossible as the introduced noise significantly affects the results^8^.

Ovaska and Valiviita reviewed in ref. ^9^ alternative approaches with a particular focus on noise amplifying effects and real-time capability of the analysed approaches. They especially noted that indirect measuring methods (e.g. numerical differentiation) require an adapted post-processing method. Further, the maximum error depends on the required acceleration bandwidth and the acceptable phase delay. Finally, they concluded that it will be important to develop direct measurement methods that do not limit the motion of the object being measured.

To overcome the difficulties of numerical differentiation, various approaches have been used via direct and indirect measuring systems in various application fields^10–12^. Padgaonkar and colleagues^10^ used a direct measuring method, the so-called nine accelerometer package (NAP), to analyse the angular acceleration signal. They equipped a dummy head with nine accelerometers to measure the acceleration state and drew conclusions about possible injuries to the brain during a simulated car crash. Their approach eliminated the need for numerical differentiation of the angular velocity vector. For a similar application, Martin and colleagues^11^ used a configuration, the so-called angular rate sensor cube (ARS), consisting of three three-dimensional accelerometers and one gyroscope, with the latter used to separately measure the angular velocity vector. This combination allowed them to calculate the angular acceleration vector in two redundant ways. Ho and Lin^12^ aimed to calculate the kinematic state of a rigid body using a similar approach to Padgaonkar and colleagues^10^. They used four three-dimensional accelerometers but no gyroscopes to measure angular acceleration. Their algorithm to calculate the angular velocity vector using rigid body equations along with only linear acceleration measurements led to equations with sign ambiguity.

One main aspect of the direct measuring approaches described in the previous work is that the individual sensors need to be fixed separately at various locations of the body segment of interest. Such approaches would not be feasible for accurate motion analysis in humans due to the placement of the sensors to the non-rigidity of body segments incorporating inevitable displacements and misalignments between sensors caused by soft tissue artefacts and the wobbling of the human body. Further, to the best of our knowledge, there is no sensor available to date that can be attached to a human body segment and can measure angular acceleration without using numerical differentiation or filtering methods.

Regarding the use of sensor fusion or a filtering algorithm, the approaches used reveal that directly measuring angular acceleration state avoids employing Kalman filters or complementary filters. There is no approach that combines a numerically derived angular acceleration signal with a directly measured one, although in theory the former would result in a more accurate signal. Published work used Kalman filters when the sensor, for instance, consists solely of accelerometers to estimate angular velocity^13^. By leveraging the known relationships (the model equations) between angular velocity and angular acceleration, the angular velocity state is estimated using a Kalman filter. In this context, there are published methods that estimate the angular acceleration state solely from position information^14^, or measure angular velocity and linear acceleration data^15^ employing Kalman filters.

From an applied perspective, an accurate assessment of kinematic quantities in monitoring human motion can provide new insights into the complex interactions between active lifestyles and increased exposure to situations in which actual loss of balance occurs. In particular, the assessment of trunk and upper body dynamics during daily-life walking is of great importance in the detection of the diminished balance control and fall risk that occurs with ageing^16–18^. The trunk comprises approximately half of the total body mass and is characterised by large lever arms from its centre of mass to the axes of rotation of the hip and ankle joint. Due to such geometric characteristics and high trunk contribution to the whole-body angular momentum during walking^19^, accurate knowledge of trunk kinematic signals is extremely important. For example, the extent to which forward-directed fall initiations during perturbed locomotion are withstood may be revealed from the regulation of trunk dynamics and whole-body angular momentum^16,20,21^. Whole-body angular momentum quantifies the balance of momenta of body segments in relation to their combined centre of mass and is close to zero during unperturbed locomotion^22^. Thus, measurement of trunk dynamics can be important in detecting failures of control during perturbed walking and may be linked to diminished balance recovery responses and falls.

In general, the use of wearable sensors for monitoring body segment kinematics in daily life, along with enhanced evaluation algorithms, has proven to be an effective approach for fall detection. The literature contains numerous approaches that demonstrate automatic fall detection, such as those proposed by Bourke and Lyons^23^, Wu^24^, Iguchi and colleagues^25^, Solaz and colleagues^26^, and Lim and colleagues^27^. All these approaches utilise wearable sensors to monitor the kinematic data of human body segments. For instance, Bourke and Lyons employed a bi-axial gyroscope mounted at the trunk^23^. Solaz and colleagues and Lim and colleagues used a three-axis accelerometer, attached with elastic belts at chest or trunk levels^26,27^. On the other hand, Iguchi and colleagues^25^ used datasets measured with an IMU. One dataset involved placing the IMU at the thigh, while the other dataset comprised head kinematics data with an IMU fixed to a helmet.

These published approaches employed either simple threshold methods, machine learning techniques, or a combination of both. Threshold approaches used specific kinematic parameters or dimensionless parameters extracted from sensor data, whereas Bourke and colleagues^23^ employed identical thresholds for all analysed individuals. However, such methods can lead to inaccuracies due to the diversity of individual behaviours, resulting in less accurate fall detection. Machine learning methods^25^, on the other hand, require the labelling of data containing various types of falls and activities in daily life (ADL) patterns, which were then provided to a learning algorithm.

Furthermore, the mentioned approaches primarily focus on recognising falls in daily activity measurements. However, more challenging distinctions, such as identifying whether an external perturbation triggers a stumble without the risk of falling, or understanding how individuals adapt to repetitive perturbations, have not been addressed adequately in previous work. From our perspective, developing sensors capable of detecting a wide range of and hence enabling an evaluation of recovery performance in response to external perturbations offers distinct advantages over sensors that solely focus on detecting falls. High sensitivity in monitoring body kinematics will allow the assessment of different magnitudes of balance recovery responses to perturbations during locomotion.

We recently showed that a direct measurement approach applied in robotics reduces the influence of noise sufficiently for obtaining sensible estimations of stability criteria from the dynamics^8^. According to this mathematical principle, the current work aimed to further develop and apply the recent findings in robotics to human motion analyses. Therefore, we developed a new embedded and wearable sensor solution to monitor kinematic quantities and assess trunk dynamics during perturbed locomotion. Our wearable inertial measurement cluster (IMC) attached to a rigid base plate provides the three-dimensional kinematic state of the attached body segment for each of its sampled time steps. As noted, the unit worked without using noise-amplifying numerical differentiation and therefore did not require signal filtering. The current work comprised two research objectives: (i) to estimate the absolute error for the new IMC by computational modelling and to compare the IMC with the direct measuring approaches NAP, ARS and a numerically derived angular acceleration signal using experimental data; and (ii) to analyse anteroposterior trunk dynamics and resolve well-known adaptation phenomena^28–32^ during simulated forward falls. The latter experimental situation involved highly standardised gait perturbations during treadmill walking and less controlled conditions, i.e. perturbed overground walking simulating real-world trips more faithfully.

The results confirmed that our proposed combination of an inertial measurement cluster and evaluation framework both provides an embedded system which eliminates numerical differentiation errors in evaluating angular acceleration enables automatic assessment of anterior fall initiation and balances recovery responses to trip perturbations.

## Results

**Fig. 1:**
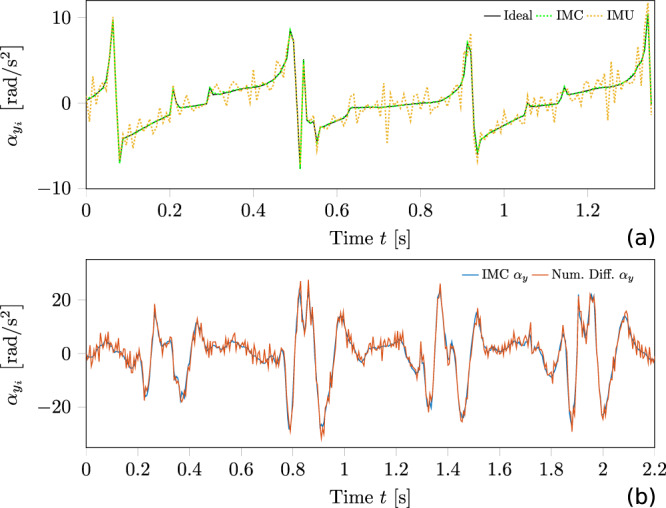
Comparison between simulated and the experimental data of the inertial measurement cluster (IMC) and the single inertial measurement unit (IMU). Graph (**a**) shows the *y*-coordinate of the robot’s trunk angular acceleration vector in simulation. The solid curve represents the ideal signal, the dotted red curve represents the noisy measurement derived from the sensor, and the dotted blue curve represents the noisy measurement of the IMU. The periodically occurring positive local maximum is a consequence of a touchdown of a foot. In (**b**), the experimental data measured by the IMC and the single IMU of the *y*-coordinate of the human being’s trunk angular acceleration vector is presented. The blue curve corresponds to the IMC signal and the red curve corresponds to the single IMU-derived signal.

By analysing and comparing the results of the simulation (simulated walking humanoid robot) and experimental data (unperturbed walking human beings), we identified similarities regarding the curve path. For example, we observed in the evaluated signal curve path a characteristic periodic pattern regarding local maxima (Fig. 1a compared with Fig. 1b and Supplementary Fig. S2). By exploiting the results of an autocorrelation analysis, we recognised a periodicity of the characteristic local maxima in both simulated and experimental data occurred with the double step frequency. These local maxima could be assigned to the touchdown event of the swing leg, as they were a consequence of the respective touchdown. The amplitudes lay in the range of approximately 8–15 rad/s^2^ for simulation data and in the range of approximately 20–40 rad/s^2^ for the experimental data.

### Computational modelling

As it is challenging to develop a method capable of applying a defined angular acceleration state, we utilised a simulation framework Webots 2022b (Cyberbotics Ltd, Lausanne, Switzerland). This solved the general equations of motion for rigid multi-body systems. As a result, it delivered the true angular acceleration vector of a rigid body as ground truth data. In this case, the simulation provided the angular acceleration vector of the trunk of the unperturbed walking humanoid robot. This type of executed movements was comparable with baseline measurements in the experimental study.

Therefore, we defined the ideal signal from the simulation framework of the angular acceleration vector as the reference signal. To estimate the accuracy of the simulated noisy IMC and IMU signals (see the section “Error analysis”), we calculated the average relative error compared to the reference signal using the root means squared error (RMSE) defined in Eq. (11). We used data from a measurement series of ten consecutive steps for this purpose. Figure 1a shows an example of the simulated measurements and the ideal angular acceleration coordinate of several steps for the transverse axis of the trunk’s principal axis system. The measured values from the IMC had a RMSE = 0.06 rad/s^2^. In comparison, the RMSE of the numerically differentiated IMU signal resulted in RMSE = 1.16 rad/s^2^.

### Experimental data

When comparing the curve path of the IMC and the numerically derived angular acceleration, it was evident that the numerically derived angular acceleration signal contained a significantly higher level of noise. We observed the highest deviations between these two signals, especially in the intervals between two touchdown events, where the angular acceleration curve path seemed to change only moderately. The mean deviation *Δ*, defined in Eq. (12), for this specific curve section, was ~105.5% from the numerically derived angular acceleration coordinate with respect to the signal evaluated by the IMC. Especially in these intervals, the IMC signal appeared to be a post-processed, filtered (noise-reduced) version of the numerically differentiated signal. The RMSE for the numerically derived signal with respect to the IMC signal was 1.68 rad/s^2^. The characteristics of the experimental data corresponded to the characteristics observed in the simulated sensor data, seen in Fig. 1. By analysing the touchdown events referring to the local maxima (higher magnitudes regarding the angular acceleration component *α*_*y*_), we observed that the relative deviations got smaller between these two approaches.

When comparing the three direct measuring approaches IMC, NAP and ARS, we noticed that the evaluated curve path of the IMC and the NAP nearly coincided (Supplementary Fig. S2). The RMSE for the NAP signal with respect to the IMC signal was 0.1 rad/s^2^ and for the ARS signal with respect to the IMC signal was 0.78 rad/s^2^. Compared to NAP and IMC, the ARS approach showed in general a greater deviation. This behaviour was mostly evident in the curve section containing higher gradients within touchdown events (Supplementary Fig. S2).

For the curve section of the perturbed gait pattern, we observed similar characteristics as in the unperturbed gait pattern evaluation. With higher magnitudes in the curve path, the relative deviation between all compared approaches in the *α*_*y*_-signal got smaller. For example, the mean deviation Δ of the local maxima and minima (regarding local extrema with absolute values exceeding ±50 rad/s^2^) in Supplementary Fig. S3 between IMC compared to the numerically derived signal was 10.3% and from IMC to ARS 4.48%.

The comparison of the three approaches considering the capability to analyse the angular velocity vector (Supplementary Fig. S4) obviously showed a conceptual advantage for the IMC and ARS approaches compared to the NAP. The directly measured angular velocity vector coordinate *ω*_*y*_ of IMC and ARS nearly coincided. In comparison with them, the angular velocity vector coordinate *ω*_*y*_ evaluated by NAP exhibited high deviations over the whole curve path.

### Repeated treadmill-based gait trip-like perturbations

**Fig. 2:**
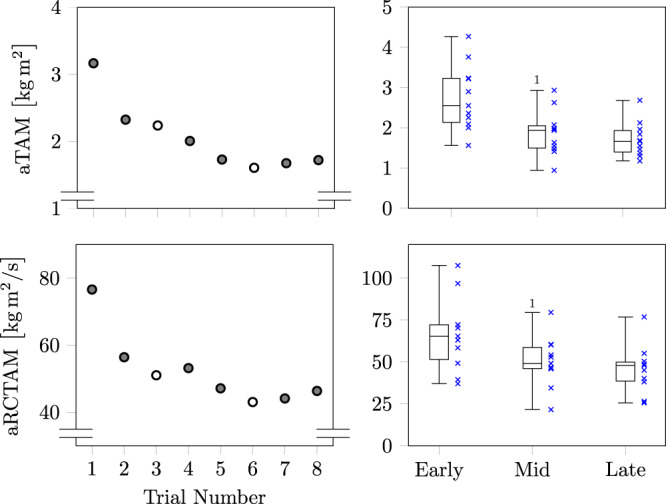
Values of the accumulated relative trunk angular momentum (aTAM) and the accumulated rate of change in relative trunk angular momentum (aRCTAM) for treadmill trip perturbations. The results averaged across all subjects for each respective trial, as well as the same data as stages in adaptation: early (trials 1 and 2), mid (trials 4 and 5), and late (trials 7 and 8) using box plots are shown. In each box plot the lower whisker corresponds to the smallest value, the upper whisker to the largest value and the box is formed by the median and the 25% and 75% quartiles.

By using our approach to automatically detect trip-like perturbations during treadmill walking, the approach identified all valid induced perturbations over the 30 min recordings in all analysed participants. The approach also defined the corresponding observation windows of the balance recovery response, indicating the onset and offset of gait pattern changes compared to unperturbed walking. There was a main effect for the time of recovery across the test period $[F(1.507,15.067)=7.117,p=0.005,{{\eta }_{p}}^{2}=0.416]$, with statistically significant lower values for mid (*p* = 0.017; 1.39 ± 0.28 s) and late (*p* = 0.048; 1.30 ± 0.25 s) adaptation phases compared to early (1.67 ± 0.34 s; Fig. 2). Within the testing period, the accumulated relative trunk angular momentum showed a monotonic reduction with repeated practice $[F(1.312,13.209)=26.213,p \, < \, 0.001,{{\eta }_{{\rm {p}}}}^{2}=0.724]$, i.e. lower values for mid (*p* = 0.001) and late (*p* = 0.001) phases compared to the early adaptation phase. The same pattern was observed for the rate of change in relative trunk angular momentum $[F(1.203,12.031)=15.069,p=0.002,{{\eta }_{{\rm {p}}}}^{2}=0.601]$, with lower values for mid (*p* = 0.016) and late (*p* = 0.005) phases compared to the early adaptation phase. The relative changes in trunk dynamics between early and late adaptation phases were on average 32%. No statistically significant differences were detected in trunk dynamics between mid and late adaptation phases.

### Repeated overground-based gait trip-like perturbations

**Fig. 3:**
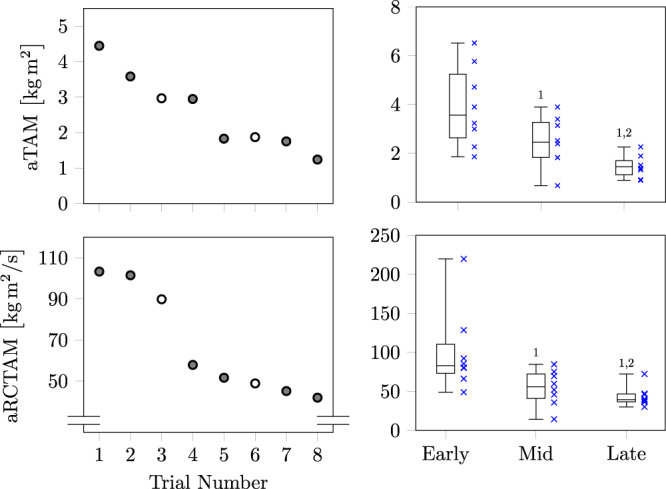
Values of the accumulated relative trunk angular momentum (aTAM) and the accumulated rate of change in relative trunk angular momentum (aRCTAM) for overground trip perturbations. The results averaged across all subjects for each respective trial, as well as the same data as stages in adaptation: early (trials 1 and 2), mid (trials 4 and 5), and late (trials 7 and 8) using box plots are shown. In each plot the lower whisker corresponds to the smallest value, the upper whisker to the largest value and the box is formed by the median and the 25% and 75% quartiles.

For overground gait perturbations, testing of two participants could not be completed due to technical issues. Furthermore, due to safety harness interference and various signal artefacts not all of the 72 perturbation trials for the remaining nine participants could be analysed, i.e sometimes only one trial was included to represent early (trial 1 or 2), mid (trial 4 or 5) or late (trial 7 and 8) phases. Accordingly, for the visualisation of the descriptive statistic in Fig. 3 we considered eight subjects providing all valid trials. Nevertheless, there was a statistically significant main effect [*χ*^2^(2) = 14.889, *p* < 0.001] for time of recovery, demonstrating lower values for mid (*p* = 0.028; 1.17 ± 0.63 s) and late (*p* = 0.021; 0.98 ± 0.57 s) in relation to the early (1.17 ± 0.84 s) adaptation phase, and for late (*p* = 0.028) compared to mid adaptation phase during overground trips. Within the period of recovery time, the accumulated relative trunk angular momentum showed continuous reductions from early to late adaptation phase [*χ*^2^(2) = 16.222, *p* < 0.001]: early vs. mid, *p* = 0.008; mid vs. late, *p* = 0.015; early vs. late, *p* = 0.008, with an average decline of ~30%. Almost the same statistical findings were obtained for the rate of change in relative trunk angular momentum [*χ*^2^(2) = 16.222, *p* < 0.001]: early vs. mid, *p* = 0.008; mid vs. late, *p* = 0.050; early vs. late, *p* = 0.008; with an average decline of ~26%.

### Lean-and-release balance recovery task

**Fig. 4:**
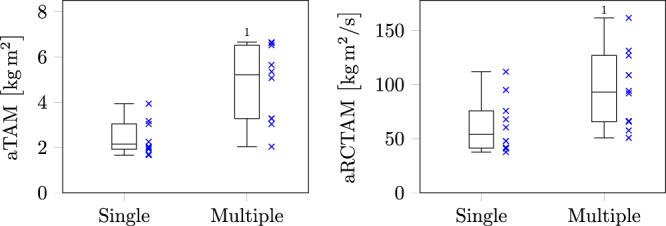
Values of the accumulated relative trunk angular momentum (aTAM) and the accumulated rate of change in relative trunk angular momentum (aRCTAM) for the lean-and-release task. The data presented represent all participants and are divided according to whether the recovery strategy involved single steps or multiple steps. In each box plot the lower whisker corresponds to the smallest value and the upper whisker to the largest value. The box is formed by the median and the 25% and 75% quartiles.

As for the overground gait perturbation task (Fig. 4), the testing of one subject could not be completed due to technical issues during the lean-and-release task. When separating the trials for each individual into single- and multiple-step recovery strategies, the time of recovery demonstrated statistically significant higher values (*p* = 0.001) for multiple versus single-stepping (1.37 ± 0.35 s vs. 0.63 ± 0.09 s). Within the identified period of recovery time, both the accumulated relative trunk angular momentum and rate of change in relative trunk angular momentum showed significantly higher values for multiple- compared to single-stepping (*p* < 0.001 and *p* = 0.005, respectively), with an average difference of ~47% and ~32%, respectively.

## Discussion

Remote monitoring using wearable sensors and accurate extraction of kinematic quantities can provide new insights into the complex interactions between active lifestyles and increased exposure to situations in which actual loss of balance occurs. Current approaches, however, are often limited to accurately extracting the body segment dynamics of functional activities. This is because they require the use of IMUs. Some kinematic quantities evaluated by IMU measurements are affected by mathematical analysis including both noise-amplifying time differentiation in relation to calculation of the angular acceleration vector and time integration to estimate the orientation. For the proposed IMC, we avoided differentiation through direct measurement of angular acceleration by combining four gyroscopes and four three-dimensional accelerometers, spatially separated and mounted rigidly on the base plate. We also added a power supply and the electronics required for the collection and processing of the measured data. This enabled real-time data acquisition and onboard processing over several hours of battery life and stand-alone use appropriate to daily life. As a first step, we assessed the IMC by investigating its use in the automated assessment of fall initiation and balance recovery responses following perturbations while walking. With the experiments, we revealed the sensitivity of the approach and the ability to enable new insights regarding the impact of anteroposterior trunk dynamics in perturbed locomotion. Due to the sensor’s wearability at human body segments and its stand-alone usability, the IMC presents an opportunity to pioneer a reliable assessment of human movement and balance in daily life.

### Comparative analysis using computational modelling and experimental data

The computational modelling analysis confirmed our hypothesis that the application of the IMC combined with a sensor fusion algorithm can significantly reduce errors caused by numerical differentiation due to the principle of direct measurement of angular acceleration. Additionally, compared to direct measuring approaches NAP and ARS by Padgaonkar and colleagues^10^ and Martin and colleagues^11^, we reduce the errors in angular velocity evaluation compared to NAP (Supplementary Fig. S4) as well as in the angular acceleration evaluation compared to ARS (Supplementary Fig. S2). The results of the simulation study demonstrate that the RMSE in angular acceleration is clearly lower when using the IMC compared to a single IMU (RMSE on average for the IMC was 0.06 and 1.16 rad/s^2^ for an IMU). Such errors introduced via numerical differentiation cannot be ignored in human movement sciences and robotics, particularly in monitoring kinematics with low ranges of angular acceleration.

To transfer the conclusions of the simulated to experimental data, we compared our approach with the direct measurement approaches NAP and ARS, as well as the numerically derived signal from a single IMU. By comparing the simulated and experimental determined IMC signals with the numerically derived signal from a single IMU, we observed nearly identical phenomena and curve path characteristics. The experimental and simulated characteristics closely correspond, indicating that we can transfer the findings of simulation-generated results to real-world applications. Finally, we revealed that the IMC did accurately resolve the true angular acceleration vector of a rigid body in experimental conditions, like what we saw in the simulation. The similarity in signal profiles for the angular acceleration coordinate between NAP and IMC supports this hypothesis. Furthermore, the experimental data revealed that our approach has advantages in the accuracy of angular velocity determination concerning NAP. This follows due to NAP needing to solve equations based on noisy linear acceleration measurements and sign ambiguity to estimate angular velocity, while our approach uses redundant direct measurements. In contrast, the ARS approach showed a more noise-prone angular acceleration signal compared to NAP and IMC. This was caused since ARS used fewer raw data signals, which makes the approach more susceptible to measurement inaccuracies in general. Moreover, our used sensor fusion algorithm reduced noise in both angular velocity and acceleration signals exploiting signal redundancy. Due to the statistical characteristic of the mathematical model for white noise, using the eight independent noise raw data signal combinations to evaluate the angular acceleration vector coordinate leads to a more accurate estimation. Regarding the use of, for example, a Kalman filter, there would not be a substantial advantage in employing a data fusion algorithm, as we aimed for a single-sensor solution using a principle of direct measurement. Also in the previous work, no Kalman filter was used for direct measurement methods^10–12^. Next, our evaluation algorithm used only the component of the angular velocity and acceleration vectors representing the anterior-posterior axis (see the section “Data evaluation framework”). Therefore, we do not need to evaluate the body segments’ orientations estimated typically by a sensor fusion algorithm, e.g., a Kalman filter.

A consequence of this dual improvement is that the IMC can resolve detail in the case of small changes in segment dynamics due to a better signal-to-noise ratio for both signals. This is particularly relevant for the automated detection of fall initiation onsets and terminations of balance recovery. Consequently, the increased accuracy of the kinematics measurements (angular velocity and acceleration vectors) of our IMC has an enormous impact on the resolvability of minimal segment dynamics. For example, in our laboratory investigations, we demonstrated it is feasible to distinguish various balance recovery responses and clearly identify well-known adaptations to perturbed locomotion^28–32^. Since our IMC is wearable (compared to NAP and ARS) and through its stand-alone usability, which allows maximally unimpaired motion of the participant, it has the potential for accurate and remote monitoring of human movement and for direct and sensitive assessment of trunk dynamics.

### Experimental assessment of perturbed locomotion in humans

One challenge for detecting anterior fall initiation is that unperturbed gait patterns and trunk dynamics differ significantly between individuals. The range of magnitudes regarding local maxima for the coordinates of angular velocity and angular acceleration around the transverse axis corresponding to touchdowns during unperturbed overground walking were 0.499–1.343 rad/s and 28.626–70.744 rad/s^2^ for the analysed participants, respectively. To address these variations objectively, we referenced our proposed threshold algorithm for the detection of anterior fall initiation and balance recovery to automatically and individually determined thresholds that were extracted from unperturbed walking curves. The advantage of our current approach is that we consider individual variations in trunk dynamics during walking via recording of reference curves during unperturbed walking. Similarly, with the flexibility gained by the threshold algorithm, we could determine personalised thresholds for diverse movements or tasks to improve the accuracy of the fall initiation detection and recovery performance analysis. Furthermore, we used the proposed quantities accumulated relative trunk angular momentum and accumulated rate of change in relative trunk angular momentum (see the section “Recovery performance evaluation”) to analyse the recovery performance and resolve adaptations. This will enable long-term comparison and benchmarking between different test subjects due to the proposed quantities combining measured absolute kinematics with the subject-specific anthropometric quantities. In our case, we weighted the measured kinematic signals with the individual subject’s moment of inertia of the trunk about the transverse axis (corresponds to *y*-axis of the trunk's principal axis) $\phantom{-}^{{{\rm{EF}}}}{\Theta }_{yy}^{(q)}$. Here, EF is the principal axes reference system of the trunk and represents the coordinate frame in which the moment of inertia is expressed. $\phantom{-}^{{{\rm{EF}}}}{\Theta }_{yy}^{(q)}$ contains the trunk mass and the geometric dimension as parameters and further the reference point *q* (midpoint of the trunk lying on the level between both spina illiaca anterior).

Previous investigations using time-consuming acquisition and data processing approaches in laboratory settings have shown that optical motion capture systems can assess adaptive improvement in balance recovery responses to repeated trip-like perturbations^28–31^. These responses involve effective increases in step length that are aimed at decreasing the distance between the anterior boundary of the base of support and the state of the centre of mass^33,34^. Another essential component of withstanding forward-directed fall initiations is the regulation of trunk dynamics and whole-body angular momentum^16,20,21^.

Using our wearable IMC located at the trunk, we were able to confirm optical motion capture studies showing that humans can limit the consequences of trip-like perturbations with repeated practice. These adaptations were observed for both repeated treadmill trip perturbations and the tested overground trips which may reflect more realistic real-life scenarios. There were continuous reductions in accumulated relative trunk angular momentum of ~30% from the early to late adaptation phase. Moreover, we were able to confirm that our IMC and its evaluation algorithm are sensitive enough not only to assess adaptational phenomena but also to distinguish between single- and multiple-stepping strategies when investigating simulated anterior falls. Notably, balance recovery performances after a sudden forward fall in a lean-and-release protocol, i.e. the ability to recover balance with a single step, can predict fall risk for older adults^35^. Thus we concluded that a failure to control relative trunk angular momentum during perturbed walking is linked to diminished balance recovery responses and that the IMC plus evaluation algorithm can be used to objectively assess anterior balance dysfunction and fall risk.

Regarding the argument that the numerically differentiated signals extracted by a single IMU would be sensitive enough to detect the balance recovery responses and resolve adaptation phenomena, we want to highlight two facts. First, a comparison of the simulation and experimental results between the IMC and single IMU-derived signal showed the present accuracy deviations considering the magnitudes and curve path characteristics (Fig. 1a, b, and Supplementary Fig. S3). Especially in the low range of angular acceleration magnitudes, the deviations are not negligible concerning balance recovery response detection. Next, physically useful results cannot be derived from it under any circumstances, as shown in ref. ^8^. Secondly, we compared the extraction of personalised threshold values from the baseline curves for all subjects with experimental data, utilising both the IMC measurements and the numerically differentiated signals from the IMU. As described in the section “Data evaluation framework”, the automated balance recovery response detection algorithm extracted the averaged local maximum and minimum and the global maximum and minimum threshold.

The detection algorithm employed the scaled global maximum and minimum thresholds to identify anomalies in the signal that exceed the baseline’s magnitude. Next, by using the averaged local maximum (referring to the touchdowns), the algorithm analysed when these characteristic local maxima of the perturbed curve path returned within the bandwidth of the averaged local and global maximum and minimum, respectively, of the baseline. This indicated the recovery of the gait pattern. We observed that the thresholds determined through numerical differentiation were notably higher than those derived from the IMC assessment. Averaging all subjects for overground walking, the global maximum threshold from numerical differentiation exceeded the IMC threshold by 29.58 rad/s^2^. Similarly, a discrepancy of 10.53 rad/s^2^ was observed for the averaged local maximum threshold (Supplementary Fig. S5). This directly leads to a reduced accuracy in detecting balance recovery responses using numerically differentiated signals. Conversely, these findings confirm that the direct measurement approach regarding angular acceleration is more sensitive.

It is important to elaborate on how the algorithm distinguishes between fall initiation due to tripping and daily life motor tasks requiring high relative trunk angular momentum such as picking up an object. Since our current algorithm evaluates the angular velocity and angular acceleration signal simultaneously, we are confident that the system could distinguish between a controlled voluntary trunk motion from a reactive trunk motion involved in stability recovery. If only the trunk angular velocity signal was measured, the lift and trip actions likely resulted in similar amplitude characteristics of the signal’s curves. An advantage of simultaneously evaluating trunk angular velocity and trunk angular acceleration signals is that lift and trip discriminations become feasible. We have provided an example data set (Supplementary Fig. S6) showing that the amplitudes of local extrema and the frequency spectrum in the curve path of the angular acceleration vector transverse coordinate are much smaller for picking and dropping (controlled voluntary trunk motion) an object compared to a trip-like perturbation. In our case, it was essential to evaluate both kinematic signals simultaneously in order to provide a robust analysis for remote and automated monitoring of anterior fall initiation and balance recovery responses.

It should be acknowledged that remote monitoring over several days may be restricted by the current size of the IMC. A shorter distance between the sensor packages will be beneficial but is likely to increase noise due to the inversely proportional characteristics regarding the distance between the sensor packages (see Eq. (10)). We also need to investigate how the typical positioning and alignment precision for the sensor packages affects miniaturised prototypes in terms of measurement accuracy. To address the above issues, further developments are required to enable the practical application of the current sensor solution in clinical or gerontological settings.

In conclusion, our proposed combination of an inertial measurement cluster and evaluation framework provides a new, embedded system which eliminates numerical differentiation errors for the evaluation of angular acceleration and enables automatic assessment of anterior fall initiation and balance recovery responses to perturbations. Due to its stand-alone usability over the course of a day, we provide new possibilities for accurate remote monitoring in human movement science, particularly for objective assessments of balance dysfunction and fall risk in designated population groups.

## Methods

### Inertial measurement cluster

#### General equations

In contrast to the use of a standard IMU, our IMC measures the angular acceleration vector directly. The unit incorporates four sensor packages, each consisting of one three-dimensional gyroscope and one three-dimensional linear accelerometer within a rigid wearable structure. It derives the angular acceleration vector using the laws of rigid-body motion and an algebraic sensor fusion algorithm. The sensor packages are placed spatially defined on a rigid three-dimensional base. The following equation applies to the acceleration of an arbitrary fixed point on a rigid body

1 $${{{{{{{{\boldsymbol{a}}}}}}}}}_{i}={{{{{{{{\boldsymbol{a}}}}}}}}}_{0}+\dot{{{{{{{{\boldsymbol{\omega }}}}}}}}}\times {{{{{{{ r}}}}}}}_{i0}+{{{{{{{\boldsymbol{\omega }}}}}}}}\times ({{{{{{{\boldsymbol{\omega }}}}}}}}\times {{{{{{{ r}}}}}}}_{i0}).$$

Here ***a***_*i*_ indicates the linear acceleration at the spatial point *i*, *r*_*i*0_ is the relative vector from sensor package 0 to sensor package *i*. ***ω*** is the angular velocity and $\dot{{{{{{{{\boldsymbol{\omega }}}}}}}}}$ the angular acceleration vector which are both constant for all points on a rigid body. The system of the coordinate equations from Eq. (1) is under-determined due to the skew-symmetric property of the cross-product. To obtain a single unique solution of each coordinate equation, the three relative vectors from sensor package 0 to all others of sensor package *i* on the rigid body must be linearly independent. Due to this, the sensor packages do not lie in a spatial plane. This ensures that the three vector coordinates are unambiguously resolvable. In general, three linear-independent vectors can form a basis of the three-dimensional Euclidean space ${{\mathbb{E}}}^{3}$ with ${{{{{{{\mathcal{G}}}}}}}}={{{{{{{{\boldsymbol{g}}}}}}}}}_{1}={{{{{{{ r}}}}}}}_{10},{{{{{{{{\boldsymbol{g}}}}}}}}}_{2}={{{{{{{ r}}}}}}}_{20},{{{{{{{{\boldsymbol{g}}}}}}}}}_{3}={{{{{{{ r}}}}}}}_{30}$. ${{{{{{{{\mathcal{G}}}}}}}}}^{{\prime} }={{{{{{{{\boldsymbol{g}}}}}}}}}^{1},{{{{{{{{\boldsymbol{g}}}}}}}}}^{2},{{{{{{{{\boldsymbol{g}}}}}}}}}^{3}$ is the dual basis of ${{{{{{{\mathcal{G}}}}}}}}$, defined by ${{{{{{{{\boldsymbol{g}}}}}}}}}_{i}\cdot {{{{{{{{\boldsymbol{g}}}}}}}}}^{j}={\delta }_{i}^{j}$. We can calculate the contravariant basis vectors of the dual basis ${{{{{{{{\mathcal{G}}}}}}}}}^{{\prime} }$ using the common calculation

2 $${{{{{{{{\boldsymbol{g}}}}}}}}}^{k}=\frac{1}{g}{{{{{{{{\boldsymbol{g}}}}}}}}}_{i}\times {{{{{{{{\boldsymbol{g}}}}}}}}}_{j},$$

where *g* = (***g***_1_ × ***g***_2_) ⋅ ***g***_3_ applies. It is well known that the contravariant basis vectors also form a basis of ${{\mathbb{E}}}^{3}$. Thus, these contravariant vectors are also linearly independent (see, e.g., ref. ^36^).

Starting by rearranging Eq. (1) to

3 $$\dot{{{{{{{{\boldsymbol{\omega }}}}}}}}}\times {{{{{{{ r}}}}}}}_{i0}={{{{{{{{\boldsymbol{a}}}}}}}}}_{i}-{{{{{{{{\boldsymbol{a}}}}}}}}}_{0}-{{{{{{{\boldsymbol{\omega }}}}}}}}\times ({{{{{{{\boldsymbol{\omega }}}}}}}}\times {{{{{{{ r}}}}}}}_{i0}).$$

we subsequently used the circular permutation of the vector triple product to separate the term $\dot{{{{{{{{\boldsymbol{\omega }}}}}}}}}$. For this purpose, the scalar product of Eq. (3) and the relative vector *r*_*j*0_ is formed as follows:

4 $$(\dot{{{{{{{{\boldsymbol{\omega }}}}}}}}}\times {{{{{{{ r}}}}}}}_{i0})\cdot {{{{{{{ r}}}}}}}_{j0}=({{{{{{{{\boldsymbol{a}}}}}}}}}_{i}-{{{{{{{{\boldsymbol{a}}}}}}}}}_{0}-{{{{{{{\boldsymbol{\omega }}}}}}}}\times ({{{{{{{\boldsymbol{\omega }}}}}}}}\times {{{{{{{ r}}}}}}}_{i0}))\cdot {{{{{{{ r}}}}}}}_{j0}.$$

Here, *r*_*j*0_ is the relative vector from sensor package 0 to sensor package *j*. Upon applying the circular permutation in Eq. (4), this gives

5 $$({{{{{{{ r}}}}}}}_{i0}\times {{{{{{{ r}}}}}}}_{j0})\cdot \dot{{{{{{{{\boldsymbol{\omega }}}}}}}}}=({{{{{{{{\boldsymbol{a}}}}}}}}}_{i}-{{{{{{{{\boldsymbol{a}}}}}}}}}_{0}-{{{{{{{\boldsymbol{\omega }}}}}}}}\times ({{{{{{{\boldsymbol{\omega }}}}}}}}\times {{{{{{{ r}}}}}}}_{i0}))\cdot {{{{{{{ r}}}}}}}_{j0}.$$

Through applying Eq. (2), using the definition of the basis ${{{{{{{\mathcal{G}}}}}}}}$, and multiplying the inverse of *g* to Eq. (5), the following results immediately:

6 $${{{{{{{{\boldsymbol{g}}}}}}}}}^{k}\cdot \dot{{{{{{{{\boldsymbol{\omega }}}}}}}}}=\frac{1}{g}({{{{{{{{\boldsymbol{a}}}}}}}}}_{i}-{{{{{{{{\boldsymbol{a}}}}}}}}}_{0}-{{{{{{{\boldsymbol{\omega }}}}}}}}\times ({{{{{{{\boldsymbol{\omega }}}}}}}}\times {{{{{{{ r}}}}}}}_{i0}))\cdot {{{{{{{ r}}}}}}}_{j0}.$$

With Eq. (6), we can calculate the covariant components of $\dot{{{{{{{{\boldsymbol{\omega }}}}}}}}}$ directly. By using the definition of the dual basis, it applies

7 $${{{{{{{{\boldsymbol{g}}}}}}}}}^{k}\cdot \dot{{{{{{{{\boldsymbol{\omega }}}}}}}}}={{{{{{{{\boldsymbol{g}}}}}}}}}^{k}\cdot {\alpha }^{i}{{{{{{{{\boldsymbol{g}}}}}}}}}_{i}={\alpha }^{k}.$$

The coordinates *α*^*k*^ of the angular acceleration vector are expressed in the covariant basis.

#### Sensor setup

**Fig. 5:**
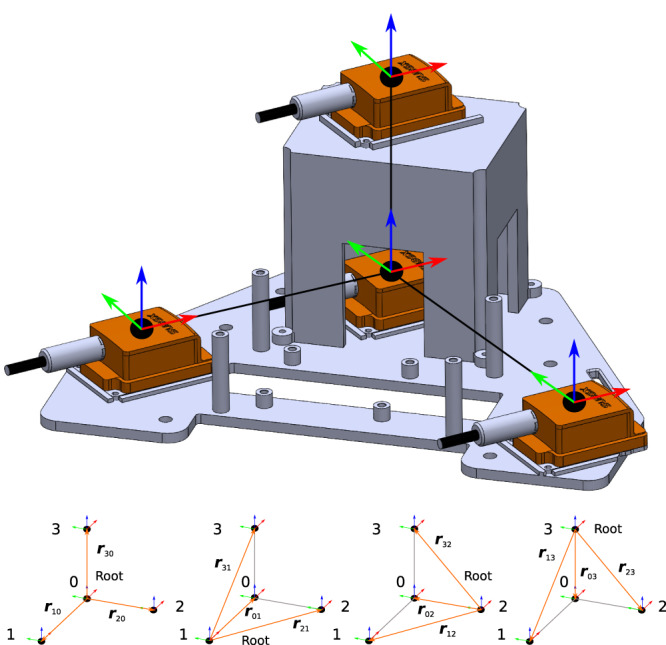
Definition of the vectors and frames used for the kinematic description of the rigid multi-body system. The shown coordinate frames of the sensor packages represent the sensor evaluation frame. The four configurations shown are the basis of evaluation for the sensor fusion algorithm.

To evaluate mechanically interpretable coordinates, we can define an arbitrary coordinate frame and transform the vector equation to its corresponding system of coordinate equations. One example is the body-fixed sensor frame. Figure 5 shows a schematic of the setup illustrating the sensor’s own evaluation frames and the four precisely defined spatial mounting points of the sensor packages. These orthogonal frames represent the so-called sensor evaluation frames in which the coordinates of the linear acceleration and the angular velocity vectors are measured.

We placed three out of the four sensor packages on top of the base plate. One sensor package is placed centrally at the upper edge of the base plate and designated with the number 0 (Fig. 5). Two further sensor packages (1 and 2, Fig. 5) were placed so that the vectors between them and sensor package 0 enclosed a 90° angle. It was thus possible to use a symmetrically constructed base plate that was comfortable to wear. For positioning the fourth sensor package (3, Fig. 5) we fixed a bracket to the base plate and aligned it directly above the sensor package 0 (Fig. 5). The relative vector *r*_30_ between these two sensor packages (0 and 3, Fig. 5) was parallel to the normal vector of the base plate. Each relative vector was thereby collinear with one basis vector of the sensor package’s own evaluation frame. The norms of the applied relative vectors for the IMC used are in this study: *r*_10_ = 0.1375 m, *r*_20_ = 0.175 m and *r*_30_ = 0.1 m. We milled out the base of a rigid polyvinyl chloride plate, with the resulting weight of the IMC prototype being ~0.8 kg.

#### Sensor fusion of redundant signals

In contrast to previous approaches, e.g. by Padgaonkar and colleagues^10^ using nine one-dimensional accelerometers and Martin and colleagues^11^ using one three-dimensional gyroscope and three three-dimensional accelerometers, we utilised four sensor packages (four three-dimensional gyroscopes and four three-dimensional accelerometers), resulting in the redundancy of the sensor signals. Therefore, we used the four separate angular velocity vectors in our algorithm and all of the measured three-dimensional linear acceleration vectors at four different points. This redundancy enables a fusion of the measured kinematics to estimate a more accurate angular acceleration vector as well as a more accurate angular velocity vector. Due to the chosen spatial structure of the sensor packages, there are four different and independent configurations for the evaluation of the angular acceleration vector. For each independent configuration, one specific sensor package was defined as the root (lower part of Fig. 5). In this way the relative vectors *r*_*ij*_ changed depending on the chosen configuration. Thus, our evaluation algorithm calculated each coordinate of the angular acceleration vector expressed in the orthogonal evaluation frame in eight independent ways. Using a sensor fusion algorithm for our IMC, we evaluated these eight independent values per vector coordinate in relation to each other to identify their validities and exclude measurement anomalies. We used the eight independent calculated scalar values for one vector coordinate to identify outliers caused by measurement inaccuracies. Therefore, we sorted the eight scalar vector coordinates. After sorting them, the algorithm selected the first value (lower limit) and the eighth value (upper limit). By identifying them, we used a weighted mean calculation principle to reduce the noise. We assigned the six remaining scalar values a high confidence and calculated the mean value of the vector coordinate with the weight *beta* (quantitatively set to *β* = 0.8). The outlier values are added separately to the mean calculation with the weight of (1−*β*). Analogue, we used the same principle to calculate a weighted mean value for the angular velocity vector coordinates considering the four redundant measured angular velocity vectors. For this, we used the weight *β* = 0.6.

#### Error analysis

There are three different categories of errors in evaluating the angular acceleration when using the IMC: (i) model assumptions; (ii) inaccuracies due to manufacturing and assembling of the IMC components; and (iii) noise behaviour of the gyroscopes and linear accelerometers themselves. Inaccuracies for (i) may have arisen from the assumption that both the IMC and the human upper body behaved like rigid bodies in the investigated motion. In our computational modelling, we have not considered inaccuracies of type (i). For inaccuracies (ii) due to manufacturing and assembling, we considered misalignment and mispositioning of the four sensor packages used. We assumed that each sensor package’s own evaluation frame had a misalignment with respect to the chosen evaluation frame after mounting the sensor packages on the base plate. In general, mispositioning meant that there was a three-dimensional offset vector Δ*r*_*i*0_ added to the desired relative vector ${{{{\bar{{{{ r}}}}}}}}_{i0}$. For (iii), we considered white noise behaviour of the gyroscopes and linear accelerometer signals for angular velocity and linear acceleration. We described the white noise of the gyroscope and linear accelerometer signals mathematically as normally distributed random errors. Thus $s(t)=\bar{s}(t)+n(t)$ generally applied. $\bar{s}(t)$ was the real but generally unknown value and *n*(*t*) was the error through white noise. We estimated *n*(*t*) for each sample time point depending on the standard deviation *σ* and the mean *μ* of the gyroscopes and linear accelerometers used. We calculated with $\sigma =\nu \sqrt{f}$ the standard deviation for the gyroscope and accelerometer as mentioned in the data sheet of Xsens MTi-20 VRU (Movella Inc., Nevada, USA), where *ν* was the noise density, and *f* was the sample rate applied. The mean *μ* was set to zero because of the white noise characteristic.

We identified one main impact regarding the accuracy by considering Eq. (6). We noticed that it contains the relative vectors. Consequently, assuming noisy sensor signals, the distances of the chosen vectors *r*_*i*0_ influence the accuracy of the whole calculation. For example, we set for the measured linear acceleration vector ${{{{{{{{\boldsymbol{a}}}}}}}}}_{i}={\bar{{{{{{{{\boldsymbol{a}}}}}}}}}}_{i}+{{{{{{{\boldsymbol{n}}}}}}}}$. Further, because we arranged the sensor packages in an orthogonal evaluation frame (EF) (Fig. 5), we got an orthonormal basis ${{{{{{{\mathcal{G}}}}}}}}={{{{{{{{\boldsymbol{e}}}}}}}}}_{1},{{{{{{{{\boldsymbol{e}}}}}}}}}_{2},{{{{{{{{\boldsymbol{e}}}}}}}}}_{3}$ with the relative vectors *r*_10_ = *r*_10_***e***_1_, *r*_20_ = *r*_20_***e***_2_, and *r*_30_ = *r*_30_***e***_3_. In this case 1, 2, and 3 refer to the *x*-, *y*-, and *z*-axis of EF. By expressing Eq. (6) with respect to EF, we get the following equation:

8 $${r}_{i0}{r}_{j0}{{{{{{{{\boldsymbol{e}}}}}}}}}_{k}\cdot \dot{{{{{{{{\boldsymbol{\omega }}}}}}}}}=({{{{{{{{\boldsymbol{a}}}}}}}}}_{i}-{{{{{{{{\boldsymbol{a}}}}}}}}}_{0}-{{{{{{{\boldsymbol{\omega }}}}}}}}\times ({{{{{{{\boldsymbol{\omega }}}}}}}}\times {{{{{{{ r}}}}}}}_{i0}))\cdot {{{{{{{ r}}}}}}}_{j0}.$$

To calculate the *k*th coordinate of the angular acceleration vector expressed in EF it applies

9 $${{{{{{{{\boldsymbol{e}}}}}}}}}_{k}\cdot \dot{{{{{{{{\boldsymbol{\omega }}}}}}}}}=\frac{({{{{{{{{\boldsymbol{a}}}}}}}}}_{i}-{{{{{{{{\boldsymbol{a}}}}}}}}}_{0}-{{{{{{{\boldsymbol{\omega }}}}}}}}\times ({{{{{{{\boldsymbol{\omega }}}}}}}}\times {{{{{{{ r}}}}}}}_{i0}))\cdot {{{{{{{{\boldsymbol{e}}}}}}}}}_{j}}{{r}_{i0}}.$$

Here, ${{{{{{{{\boldsymbol{e}}}}}}}}}_{k}\cdot \dot{{{{{{{{\boldsymbol{\omega }}}}}}}}}$ are the coordinates of the angular acceleration vector $\dot{{{{{{{{\boldsymbol{\omega }}}}}}}}}$ expressed in EF. *r*_*i*0_ is defined as *r*_*i*0_***e***_*i*_. ***e***_*i*_ is one basis vector of the orthogonal evaluation frame and *r*_*i*0_ is the coordinate describing the distance between sensor package 0 to *i*. By showing the general equations for an arbitrary basis in the section “General equations”, we proved, without loss of generality, that the general equations are valid to calculate the angular acceleration vector despite non-orthogonal coordinate frames caused by mispositioning and misalignment of the sensor packages.

Especially, if we let the distances *r*_*i*0_ tend to zero the mounting positions of those sensor packages would converge. This would lead to that the real linear acceleration vector ${\bar{{{{{{{{\boldsymbol{a}}}}}}}}}}_{i}$ converging towards ${\bar{{{{{{{{\boldsymbol{a}}}}}}}}}}_{0}$ whereas the noise behaviour ***n*** and consequently its contribution to the errors of the measurement would remain unaffected through the spatial shifting. After inserting the above-mentioned mathematical noise definition in Eq. (9), we see it contains terms like

10 $$\frac{({{{{{{{{\boldsymbol{a}}}}}}}}}_{i}-{{{{{{{{\boldsymbol{a}}}}}}}}}_{0})\cdot {{{{{{{{\boldsymbol{e}}}}}}}}}_{j}}{{r}_{i0}}=\frac{({\bar{{{{{{{{\boldsymbol{a}}}}}}}}}}_{i}+{{{{{{{{\boldsymbol{n}}}}}}}}}_{i}-({\bar{{{{{{{{\boldsymbol{a}}}}}}}}}}_{0}+{{{{{{{{\boldsymbol{n}}}}}}}}}_{0}))\cdot {{{{{{{{\boldsymbol{e}}}}}}}}}_{j}}{{r}_{i0}}.$$

The noise behaviour ***n***_*i*_ and ***n***_0_ of the gyroscopes and linear accelerometers and the magnitude of the norms of the relative vectors *r*_*i*0_ influence the calculated angular acceleration coordinates. Consequently, due to the characteristics of in Eq. (10), we have tried to design the geometric dimensions of the prototype so that it fits the dimensions of the average anthropometrics of an adult’s thorax. Thereby, we have used the optimum between the wearability of the sensor and noise impact through the sensor package’s distance.

### Validation by computational modelling using a humanoid robot

As it is challenging to develop a method capable of applying a defined angular acceleration state to generate experimental data, we utilised a simulation framework. In the simulation, it is possible to generate ground truth data of the angular acceleration vector from a rigid body, by solving the general equations of motion. Additionally, we can simulate the IMC sensor with typical manufacturing errors and noise behaviour (manufacturer’s specification) applied. Since we used in this work the IMC to evaluate trunk movements about the transverse axis during human bipedal locomotion, we validated the IMC on the humanoid robot in simulation (Supplementary Fig. S1). We used the simulation tool Webots 2022b (Cyberbotics Ltd, Lausanne, Switzerland). Details about the humanoid robot and the simulation model used are provided in Supplementary Note 1.

We modelled errors of the physical IMC and the single IMU affecting the accuracy of the measurement results in the simulation to ensure that the results of this validation with simulated sensors can be used as realistic indicators for real-world application. Therefore, both sensors were modelled with real-world errors including mispositioning, misalignment and white noise when using gyroscopes and linear accelerometers. To estimate the error boundaries for mispositioning and misalignment the IMC was measured using a 3D measuring machine (Zeiss Prismo Ultra, Oberkochen, Germany). The maximum error boundaries from this evaluation were used for modelling these errors in the simulator. Therefore, the sensor packages 1–3 were shifted three-dimensionally in relation to sensor package 0 (general mispositioning) and rotated further against each other (general misalignment).

The mispositioning expressed in EF was defined as $\phantom{-}^{{{\mathrm{EF}}}}\Delta {r}_{i0}=\phantom{.}^{{{\mathrm{EF}}}}{r}_{i0}-\phantom{.}^{{{\mathrm{EF}}}}{{\bar{r}}}_{i0}$. $\phantom{-}^{{{\rm{EF}}}}{{{{\bar{{{{r}}}}}}}}_{i0}$ was the real but generally unknown value. The applied magnitudes of mispositioning expressed in the sensor evaluation frame for sensor packages were ^EF^Δ*r*_10_ = [0.01 m, −0.008 m, 0.002 m]^T^, ^EF^Δ*r*_20_ = [−0.005 m, 0.003 m, −0.003 m]^T^, and ^EF^Δ*r*_30_ = [0.008 m, 0.002 m, 0.005 m]^T^.

In addition to the mispositioning of the sensor packages, we applied also a misalignment to the corresponding sensor packages 1–3. Every sensor package was misaligned in a single axis of EF. Consequently, we applied an elemental rotation ${{{{{{{{\boldsymbol{R}}}}}}}}}_{i0}(\Delta {{\phi }_{i0}}_{j})$ about one of the axes of the respective sensor package coordinate frame. The subscript index *i*0 of ***R*** indicates the concrete sensor package *i* for *i* = 1,  2,  3 to which the elemental rotation is applied with respect to sensor package 0. The subscript index *j* for *j* = *x*, *y*, *z* indicates the axis to which the misalignment was added. The magnitudes of misalignment expressed in the sensor evaluation frame were $\phantom{-}^{{{\rm{EF}}}}\Delta {{\phi }_{10}}_{x}$ = 0.017 rad, $\phantom{-}^{{{\rm{EF}}}}\Delta {{\phi }_{20}}_{y}$ = −0.035 rad, and $\phantom{-}^{{{\rm{EF}}}}\Delta {{\phi }_{30}}_{z}$ = −0.026 rad. These quantities corresponded to measurement results obtained with the 3D measuring machine.

In addition, white noise was added to each measured sensor signal as described in the section “Error analysis”. The magnitudes of the added noise density for all gyroscopes were *ν* = 5.236e^−4^ rad/s/$\sqrt{{\rm{Hz}}}$ and for all accelerometers *ν* = 6.116e^−5^ m/s$\phantom{-}^{2}/\sqrt{{\rm{Hz}}}$. For the simulated data comparison of the IMC and single IMU approaches, we took the raw measurements of sensor package 0 used in the IMC to represent the single IMU. Consequently, the same parameters for the noise behaviour concerning the gyroscope of sensor package 0 ensure optimal comparability.

### Verification by experimental data

To analyse the performance and to compare our IMC to the state of the art with experimental data, we implemented the approaches of Padgaonkar and colleagues^10^ (NAP), of Martin and colleagues^11^ (ARS), and calculated the numerical differentiation using the backward difference method. This comparison was possible because we utilised a generalised sensor setup with redundant signals. The generalized sensor setup also covered the ability of replicating previously published methods such as NAP or ARS. Accurate representation was achieved by disabling redundant sensors, ensuring that the available raw data signals matched one-to-one with the signals used in the published approaches. For the comparison of the estimated angular acceleration state between those four approaches, all of the approaches used the same sensor package raw data (four single Xsens MTi-20 VRU with a sample rate of 200 Hz) of the same torso movement as the basis to extract the angular acceleration vector. This ensured the comparability between the results of the approaches, as the raw signal quality, noise behaviour, manufacturing issues, and the ambient condition were identical. Since no ground truth data were available, we did not calculate absolute accuracy parameters in this comparison. We also compared the results of the simulation and experimental data to identify correlations and drew conclusions for use cases in practice.

### Participants and experimental set-up

Eleven healthy and moderately physically active adults were recruited (data as averages and standard deviations 29 ± 6 years of age, 1.76 ± 0.12 m height, 77.8 ± 8.8 kg body mass; nine males, two females). Exclusion criteria were any neurological or musculoskeletal injuries or impairments limiting locomotion. Upon providing consent, participants were fitted with the custom-made IMC. It was attached to their back (level of thoracic vertebrae 1–12). We used adjustable straps to match the level of attachment of the IMC to the specific anthropometrics of the subject’s trunk. Further, these straps provided stabilisation of the base plate at shoulder and waist levels hence greatly reducing relative movement between the IMC and the trunk. This allowed the measured kinematics of the IMC to represent the trunk kinematics to a close approximation. During all three tasks evoking anteriorly directed balance perturbations three-dimensional kinematics of the trunk were measured. For the current analysis of anterior perturbations, we evaluated the vector coordinate of the transverse axis in the principal axis system of the trunk. The transverse axis corresponds to the *y*-axis of the evaluation frame. Before each measurement, we calibrated the IMC. To reduce constant offset errors in the measurement series of the IMC for each sensor frame axis, we calibrated the IMC through a static calibration measurement of the IMC sensor over ~30 s. We calculated the mean over all measurement samples for each vector coordinate of the angular velocity and angular acceleration vector to reduce the impact of white noise. We defined the calculated mean value as the constant offset for the respective axis and subtracted this value at each sample point of the subsequent measurement process.

Throughout all measurements, participants wore their own non-slippery leisure/sports shoes and were protected by wearing a safety harness connected to an overhead track which allowed for full range of motion in the anterior-posterior and medio-lateral directions but prevented contact of any part of the body with the ground (except for the feet). The measurements comprised eight successive trip-like perturbations, both while walking on a treadmill and walking overground. In addition, all participants were exposed to eight to ten trials of sudden anterior balance loss from various static forward-lean angles (lean-and-release task). Measurements were reviewed and approved by the ethics committee of the School of Applied Sciences at London South Bank University (approval ID: SAS1826b) and met all requirements for human experimentation in accordance with the Declaration of Helsinki^37^.

### Perturbed locomotion analyses

#### Repeated treadmill-based gait trip-like perturbations

**Fig. 6:**
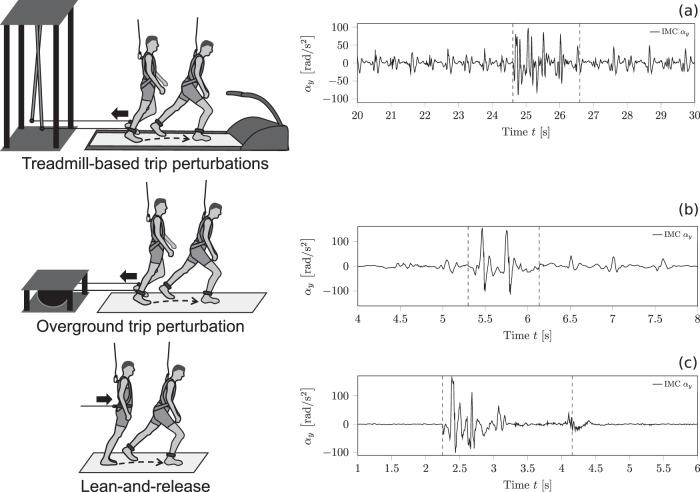
Schematic illustration of the three perturbation tasks, and respective sensor recordings. Treadmill-based trip perturbations (**a**) consisted of eight successive trip-like gait perturbations during treadmill walking. Overground-based trip perturbations (**b**), involved participants in eight successive trip-like gait perturbations while walking overground. Lean-and-release (**c**), required participants to recover stability after release from various forward-inclined positions. Safety harnesses were worn during all tasks to prevent contact of any part of the body with the ground (except for the feet). The vertical dash lines in (**a**–**c**) on the right-hand side define the observation window which was detected automatically via the threshold algorithm based on individually derived thresholds from baseline measurements.

The trip-like perturbation paradigm has been used in previous studies^28–32^. Four to seven days prior to measurements, all participants were familiarised with unperturbed treadmill walking. On the day of measurements, participants walked on a treadmill (Valiant 2 sport XL; Lode BV, Groningen, The Netherlands) at a standard speed (1.4 m/s), equipped with the IMC. A Teflon cable and ankle strap connected each of a participant’s ankles to a custom-built pneumatically driven perturbation device located behind the treadmill (Fig. 6a). Following 4 min of walking^38^, recordings of 2 min of continuous walking served to determine baseline (unperturbed) gait patterns for each participant^31^. As the participants continued to walk, eight trip-like perturbations were induced unexpectedly, with each successive perturbation being followed by a variable washout period (2–3 min) of unperturbed walking^28,31^. The perturbations were induced by activating a pneumatic cylinder using a hand trigger connected to the perturbation device. A restraining force (140 N, rise time 20 ms) was thereby applied to the left limb via a Teflon cable and ankle strap during mid-stance phase of the right foot to standardise an interruption to motion of the left limb during its mid-swing. The restraining force was released at touchdown of the left foot to allow for continuity in walking after the perturbation. Although participants were informed that their gait would be perturbed at some points during walking on the treadmill and they were encouraged to continue moving forward, the onset and removal of the resistance was applied without any immediate warning.

#### Repeated overground-based trip-like perturbations

Participants walked at a standard speed (1.4 m/s) on a custom-built flat wooden walkway (8 m length, 1.2 m width), with Teflon cables and ankle straps attached to both ankles^32,39^. The cables were in turn attached to a custom-built pneumatically driven brake-and-release device located behind the walkway (Fig. 6b). Walking speed was monitored live via an optical motion capture system that recorded a reflective marker located on the seventh cervical vertebra (16 infra-red cameras operating at 120 Hz; Miqus2, Qualisys, Gothenburg, Sweden). Once participants arrived at the end of the walkway, they were guided back to the initial position to prevent tangling of the Teflon cable. Thus only one direction of movement was considered for measurements. Following familiarisation with this walking, recordings of three consecutive forward walking trials served to determine stability control during movement on the walkway. Subsequently, eight trip-like perturbations were induced at random forward-walking trials (wash-out trials in between) but only if the standard speed was consistently reached. As for the treadmill-based task, overground gait trip-like perturbations were operated by means of a hand trigger connected to the perturbation device and evoked by a braking action of the Teflon cable on the left leg - during the mid-stance phase of the right foot and released at touchdown of the left foot. Like the treadmill-based task, perturbations were neither practised in advance nor announced immediately before exposure.

#### Lean-and-release task

This task was operated according to previous studies^40,41^. Participants were forward-inclined with their feet placed flat and at hip-width at various inclination angles so as to cover a wide range of task demands. The inclination was maintained by means of an inextensible, horizontal supporting cable attached to a belt around the participant’s pelvis and at the other end to a custom-built pneumatically driven brake-and-release system. To initiate the perturbation, the supporting cable was suddenly released within 10–30 s after the participant was stabilised in the starting position (Fig. 6c). Participants were asked and encouraged during prior task instruction, to choose the left or right leg for recovery of stability after release using a single step. Nevertheless, the inclination was varied for all participants in order to cover a range of demand and thereby to provoke minima of three single-step strategies and three multiple-step strategies for each participant. Recovery stepping strategies were classified as single or multiple stepping according to a previous description^40^. Briefly, participants were classified as single steppers if only one step was required to regain balance or if a follow-up step of the contralateral limb did not exceed the anterior displacement of the recovery limb. Accordingly, multiple stepping was defined as involving any additional step of the recovery limb or if the participant took a contralateral step exceeding the anterior displacement of the recovery limb.

### Data evaluation framework

We developed an automated threshold algorithm to detect anomalies in the time course of a measurement series. The detection of fall initiation and balance recovery and adaptations therein were based on the joint analysis of angular velocity and angular acceleration signals. The gait pattern of an individual may vary (ranges in transverse axis coordinates of angular velocity and angular acceleration touchdown-induced local maxima during unperturbed overground walking were 0.499–1.343 rad/s and 28.626–70.744 rad/s^2^ for the analysed participants, see the section “Experimental assessment of perturbed locomotion in humans”. Further, its gait pattern is influenced by the participant’s circumstances (e.g. treadmill vs. overground gait). To allow for individual gait characteristics, we applied automated threshold determination for each participant. For the determination of personalised thresholds, unperturbed gait measurements of the respective task (treadmill or overground walking) containing angular velocity and angular acceleration vectors served as a baseline measure for each participant. The respective baseline measurements involved each subject’s gait behaviour characteristics, including curve path, magnitudes of local maxima and minima, and frequency content of periodically occurring patterns. It is important to note that we measured the baseline curves with the same settings and with the same IMC sensor unit and placement of it. We did this to ensure the best possible comparability from baseline curves to perturbed gait analysis.

**Fig. 7:**
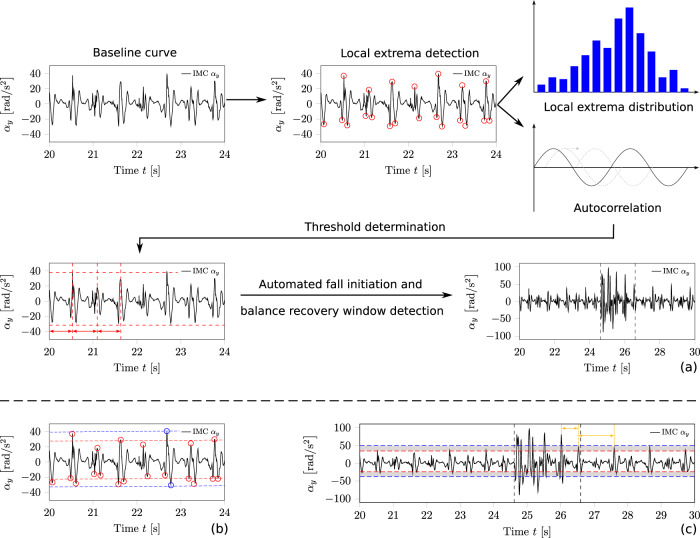
Automated threshold determination algorithm to detect balance recovery responses. The thresholds are determined individually for each participant regarding the respective task (treadmill or overground walking). Automated threshold determination analyses an unperturbed baseline curve using a combination of local extrema detection algorithm, determination of the local extrema distribution, and autocorrelation analysis, as shown in (**a**). The procedure to evaluate the averaged local maximum and minimum thresholds (red dotted lines) and the global maximum and minimum thresholds (blue dotted lines) is shown in (**b**). Red and blue circles mark the used local extrema for the respective threshold. The application of both types of thresholds to define the observation windows of balance recovery responses in the perturbed measurement series is shown in (**c**). For example, the averaged local maximum and global maximum threshold line form a bandwidth to evaluate the time point of the alignment of the perturbed curve with the baseline. The yellow arrows indicate the frequency content of the touchdown-induced local maxima.

Personalised thresholds for angular velocity and angular acceleration were derived by combining results of iteratively used local extrema detection algorithm, monitoring of local extrema value distributions, and autocorrelation analysis of the baseline measurements of each participant (Fig. 7a). Finally, the local extrema detection algorithm was able to extract both the local maxima and minima of a signal, appearance times of local extrema, and the resulting frequency spectrum over the entire measurement period. Further, we used an autocorrelation analysis to determine periodically repeating patterns in the curves and drew conclusions about, for example, the step frequency. The determined personalised thresholds were then applied to the analysis of the perturbation data series (Fig. 7a).

#### Extraction of personalised thresholds

In our detection algorithm, we used two kinds of thresholds for the analysed components of the angular velocity and acceleration vector, respectively. On the one hand, we determined the global maximum and minimum (represented through the blue circles in Fig. 7b) within the baseline curve. Therefore, we used the local extrema detection algorithm. On the other hand, we estimated the averaged local maximum of periodically occurring touchdown-induced local maxima and the averaged local minimum threshold, shown in Fig. 7b as the red dotted lines, respectively.

To determine the averaged local maximum and averaged local minimum threshold, the local extrema detection algorithm analysed each local maximum and minimum between two zero-crossings over the entire baseline curve (relevant extrema are marked with a red circle in Fig. 7b). To determine solely the touchdown-induced local maxima, the detection algorithm applied a iteratively used local maxima detection. This means that the local extrema detection algorithm worked as an iterative process in combination with local maxima distribution monitoring and autocorrelation analysis. Thereby, the local maxima distribution monitoring component subsequently divided the detected local maxima into magnitude ranges. With the occurrence count of the various local maxima magnitude ranges, this part of the algorithm eliminated the irrelevant local maxima magnitudes. Using the resulting irrelevant magnitudes as an adjusted filter, the algorithm performed again the local maxima detection process. Adjusted filter means here that the local extrema detection component of the algorithm ignored the as irrelevant defined magnitudes. In addition, by determining just the touchdown-induced local maxima the periodicity in the gait pattern could be read out. Using this, the algorithm repeated the process until the periodicity of all detected local maxima matched approximately the doubled step frequency (analysed by autocorrelation).

The averaged local minimum threshold determination worked more simply due to there was no gait pattern event corresponding to one of a local minimum periodically. Analogue, the algorithm used the local minimum distribution monitoring component to filter out the local minima with low absolute magnitudes. In contrast, this filtering process was performed once and not iteratively. This sequence of analyses and threshold determination procedures led to the definition of the threshold bandwidth, bandwidth being the span between averaged local maximum and global maximum thresholds, and averaged local minimum and global minimum thresholds seen in Fig. 7c.

#### Observation window determination

We utilised observation windows to encapsulate the balance recovery response to perturbation in the measurement series. By using data within the observation window, the algorithm evaluated the recovery performance of the participants and also registered their adaptations to repeated perturbations. A balance recovery response to perturbation during locomotion (and hence the onset and offset of an observation window) was defined by involving local maxima and minima of both angular velocity and angular acceleration of magnitudes >1.1 times the global maximum and/or minimum of the baseline curve. For this, the evaluation algorithm compared the detected local maxima or minima between two zero-crossings in the perturbed measurement curve with the scaled global maximum and minimum of the baseline curve.

If the local extrema detection algorithm detected an anomaly in the curve path for the angular velocity vector coordinate, it checked the curve path for the angular acceleration vector coordinate for the surrounding time points to verify whether or not this signal also contained an anomaly in the curve path. We defined the window of surrounding time points by the zero crossings of the local extrema classified as an anomaly in the angular velocity curve. Provided that anomalies occurred in both angular velocity and angular acceleration curves over the same observation window, we defined this as a balance recovery response to perturbation.

In order to determine the offset of the observation window, the subsequent time points after the detected anomaly were analysed. For this purpose, the algorithm used bandwidths enclosed between the averaged local maximum threshold (upper red dotted curve in Fig. 7c) and the global maximum threshold (upper blue dotted curve in Fig. 7c), the averaged local minimum threshold (lower red dotted curve in Fig. 7c) and the global minimum threshold (lower blue dotted curve in Fig. 7c) and the frequency content of the touchdown-induced local maxima (yellow arrows in Fig. 7c). We limited the time points for subsequent analysis to the time equivalent of six steps. Specifically, to detect the offset of the observation window the algorithm analysed both the angular velocity and angular acceleration curves of the perturbed measurement. The offset was defined as when the local maxima and minima of the measurement series aligned to the threshold bandwidths for the first time. As shown in Fig. 7c, in the context of bandwidth, aligned means the local extrema of the series were at least within the respective bandwidths. A separate criterion is that high-frequency content within the balance recovery response must have decayed (see the black curve in Fig. 7c) compared to the frequency content for the baseline. Quantitatively, the frequency content of the perturbed measurement series had to converge approximately to the doubled touchdown frequency to be considered as decayed. The offset of the observation window was set to the time point of the first zero-crossing following the last irregular local extrema of the angular acceleration signal. Similarly, we defined the onset of the observation window by the time point of the zero-crossing of the first local extrema of the angular acceleration measurement.

#### Recovery performance evaluation

After defining the observation window of a balance recovery response, a quantitative evaluation of the balance recovery response was performed separately via curve path characteristics of angular velocity and angular acceleration. The total duration of the balance recovery response is called the time of recovery, representing the time required to return to a stable state. We also considered integrals of the curves for both angular velocity as well as angular acceleration within the observation window to evaluate the balance recovery response. To ensure better comparability between participants, we scaled the trunk kinematics with the subject’s moment of inertia of the trunk for the transverse axis ^EF^${\Theta }_{yy}^{(q)}$. Here, EF is the principal axes reference system of the trunk and represents the coordinate frame in with the moment of inertia is expressed and *q* represents the reference point. Therefore, we could interpret the quantity ^EF^${\Theta }_{yy}^{(q)}$ ^EF^*ω*_*y*_ as the *y*-coordinate of the relative angular momentum expressed in the EF, which is defined in ref. ^42^ since the used *y*-axis is a principal axis. The quantity ^EF^${\Theta }_{yy}^{(q)}$ ^EF^*α*_*y*_ is then the rate of change in the *y*-coordinate of the relative angular momentum expressed in the EF, when we consider the planar motion of the trunk. Overall, this led to the quantities accumulated relative trunk angular momentum (${{{{{{{\rm{aTAM}}}}}}}}:={\int}_{{t}_{{{{{{{{\rm{OW}}}}}}}}}}$ ^EF^${\Theta }_{yy}^{(q)}$ ^EF^∣*ω*_*y*_∣d*t*) and accumulated rate of change in relative trunk angular momentum (${{{{{{{\rm{aRCTAM}}}}}}}}:={\int}_{{t}_{{{{{{{{\rm{OW}}}}}}}}}}$ ^EF^${\Theta }_{yy}^{(q)}$ ^EF^∣*α*_*y*_∣d*t*). We want to mention explicitly that these introduced quantities no longer have a physical interpretation. Hence, we performed numerical integration of both curves (angular velocity and angular acceleration) within the observation window under the condition that we added the area enclosed by the negative curve segments positively to the total area. We justify this since these curve segments are also an indicator of the magnitude of the balance recovery response.

To determine the total moment of inertia ^EF^${\Theta }_{yy}^{(q)}$, we measured the upper body dimensions of every participant using a motion capture system (see the section “Repeated overground-based trip-like perturbations”) and define the reference point *q* as the midpoint of the trunk lying on the level between both spina illiaca anterior. We placed markers at the seventh cervical vertebra and on left and right acromium, spina illiaca anterior and spina illiaca posterior. To estimate trunk width dimensions, we took the distance between the right and left acromion in the frontal plane. Trunk length was determined by the averaged distance from the seventh cervical vertebra to the left and right spina illiaca posterior in the sagittal plane, whereas trunk depth was defined by the averaged distance between spina illiaca anterior to posterior of the right and left side in the transversal plane. The upper body was modelled as an elliptical slab^43^.

#### Statistics

We used the simulation of the IMC attached to the trunk of a bipedal walking humanoid robot to validate the IMC. Using two angular acceleration vector signals (one from the ideal simulation result and the noisy signal from the sensor type being compared) we evaluate the accuracy of the simulated sensors with the RMSE for the IMC and IMU data points compared to the ideal result as follows:

11 $${{{{{{{\rm{RMSE}}}}}}}}=\sqrt{\frac{\Sigma {({{\alpha }_{y}}_{{{{{{{{\rm{method}}}}}}}}}-{{\alpha }_{y}}_{{{{{{{{\rm{ideal}}}}}}}}})}^{2}}{n}}.$$

A further quantitative evaluation is the relative error Δ. It is defined as

12 $$\Delta =\mathop{\sum }\limits_{i=0}^{n}\frac{| {f}_{{{{{{{{\rm{proc}}}}}}}}}({t}_{i})-{f}_{{{{{{{{\rm{ref}}}}}}}}}({t}_{i})| }{| {f}_{{{{{{{{\rm{ref}}}}}}}}}({t}_{i})| }\frac{1}{n}.$$

Here, *f*_proc_(*t*_*i*_) represents the value at the time point *t*_*i*_ of the investigated procedure’s signal. Similarly, *f*_ref_(*t*_*i*_) represents the reference signal for the comparison at the time point *t*_*i*_.

Concerning the investigations with human participants, the adaptations of participant responses from practice of perturbation tasks for both treadmill and overground walking were examined by pooling trials. Data were combined for perturbations 1–2, 4–5 and 7–8 and labelled early, mid and late adaptation, respectively. For the lean-and-release task, trials were first divided into single- and multiple-stepping strategies. The mean values across trials (separated by strategy) were calculated for each participant so as to compare analysed parameters between balance recovery strategies. Parametric assumptions for all analysed parameters (time of recovery, accumulated relative trunk angular momentum, rate of change in relative trunk angular momentum) were checked using Shapiro–Wilk tests (*p* > 0.05). Possible differences between trip-like perturbation trials (early vs. mid vs. late adaption phase) were examined for treadmill walking using one-way repeated measures analysis of variance (ANOVA) and for overground walking using separate Friedman tests. In cases of significant main effects, Bonferroni post-hoc corrections were applied. Concerning the lean and release task, *t*-tests for dependent variables were used to assess potential differences between single- and multiple-stepping recovery, and hence balance performance for each of the analysed parameters. All analyses were performed using SPSS Statistics (v27, IBM; Chicago, IL, USA) and MATLAB (2020b, MathWorks, Natick, MA, USA). If not stated otherwise, statistical significance was set at *α* = 0.05.

### Reporting summary

Further information on research design is available in the Nature Portfolio Reporting Summary linked to this article.

### Supplementary information

Supplementary information

Reporting Summary

## Data Availability

All datasets generated during and/or analysed during the current study are available on Figshare with identifier 10.6084/m9.figshare.24781506.v1.

## Code availability

The code used for validation by computational modelling, the robot, and the framework (mathematical algorithms) to analyse the kinematic data generated during the current study is being made available by the corresponding author for research (non-commercial use) or on reasonable request.
