# Supplementary material for: A wearable sensor and framework for accurate remote monitoring of human motion
Source: Commun Eng. 2024 Jan 30;3:20. doi: 10.1038/s44172-024-00168-6 (PMC11101649; doi:10.1038/s44172-024-00168-6)
Supplement: Supplementary file 1 — Supplementary information [file 44172_2024_168_MOESM1_ESM.pdf]

A wearable sensor and framework for accurate remote monitoring of human motion

Maximilian Gießler[1,2*], Julian Werth[2], Bernd Waltersberger[1] and Kiros Karamanidis[2,3]

[1*]Department of Mechanical and Process Engineering, Offenburg University of Applied Sciences, Offenburg, Germany.
[2*]Sport and Exercise Science Research Centre, School of Applied Sciences, London South Bank University, London, United Kingdom.
[3*]Department of Sport Science, Faculty of Mathematics and Natural Sciences, University of Koblenz, Koblenz, Germany.

*Corresponding author(s). E-mail(s): maximilian.giessler@hs-offenburg.de;

Supplementary information

Supplementary Note 1

Equivalent to our studies with human participants, the IMC was mounted in the simulation at the trunk of the robot for the validation investigations. The simulator solved the equations of motion for the provided bipedal gait pattern of the simulated robot Sweaty and delivered for each simulated time point the sensor measured values applied in the simulation model. Further, by using the setup of a bipedal locomotion of a humanoid robot, we validated the performance of both sensors (IMC and standard IMU) with similar motion patterns and fixing positions compared to the investigations in the experimental settings with the human subjects.

Sweaty (Figure S1), 1.65 m tall and weighing 27.6 kg, is a custom-designed, fully autonomous humanoid robot with 39 degrees of freedom. Sweaty is bipedal capable of moving omnidirectional at different speeds. These characteristics of the robot made it suitable for the validation of the IMC allowing a realistic comparison to the motion patterns of the human participants in our study. The Webots 2022b software package was used to simulate Sweaty's movements. The simulation time step was specified at 0.005 s corresponding to the sample frequencies of sensor packages used in the physical IMC for the experiments.

Sweaty's software architecture is based on Robot Operating System 2 (ROS2), transferring data in a network according to the publisher and subscriber principle. Figure S1 shows the ROS2 nodes as well as the structural connections used to generate the validation results. The interface between ROS2 and the simulator was the node called Webots-Controller-Client. Via this interface, the node Motion sent the desired walking pattern of the robot to the simulator. Models for IMC and IMU, including realistic misalignment and mispositioning errors, and the spatial mounting position at the robot's trunk were provided to the simulator. The physics engine evaluated the dynamics by solving the equations of motion for the robot as a rigid multi-body system with the IMC and IMU attached. To apply also realistic white noise behaviour, the Gaussian distributed noise was added in the Signal Processing node to each measured raw signal of the IMC and IMU. Finally, we used the noisy sensor signal outputs for the angular acceleration vector estimation of the IMC and the numerical derivative (backward difference quotient) of the single IMU.

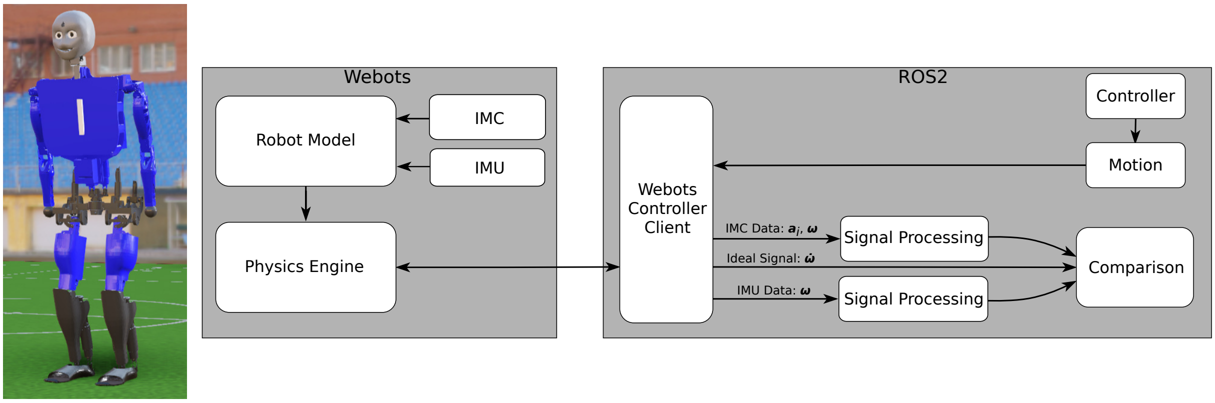

Fig. S1 Simulation model of the humanoid robot Sweaty. The graphic of the nodes and simulator showing the connections between them used to generate the simulation results to determine the capability of the inertial measurement cluster (IMC) and a standard inertial measurement unit (IMU).

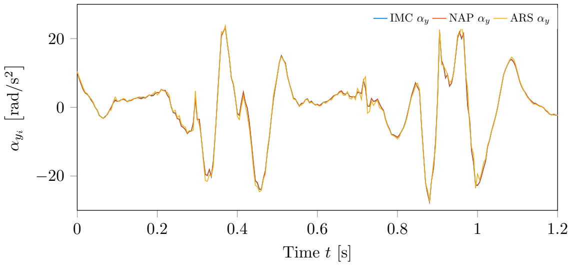

Fig. S2 Section of the curve path of angular acceleration vector coordinate of the transverse axis (principle y-axis of the trunk) during unperturbed overground walking evaluated by the inertial measurement cluster (IMC), Nine Accelerometer Package (NAP) and Angular Rate Sensor cube (ARS).

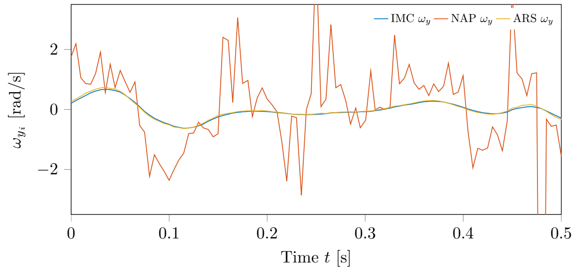

Fig. S4 Section of the curve path of angular velocity vector coordinate of the transverse axis (principle y-axis of the trunk) during unperturbed overground walking evaluated by the inertial measurement cluster (IMC), Nine Accelerometer Package (NAP) and Angular Rate Sensor cube (ARS).

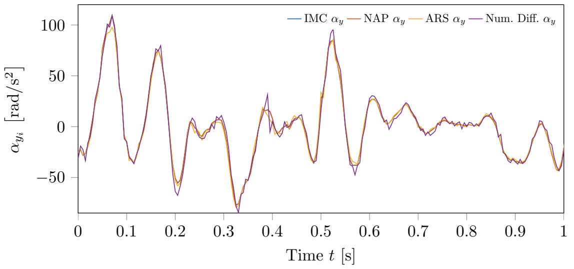

Fig. S3 Section of the curve path of angular acceleration vector coordinate of the transverse axis (principle y-axis of the trunk) during perturbed overground walking evaluated by the inertial measurement cluster (IMC), Nine Accelerometer Package (NAP) and Angular Rate Sensor cube (ARS). This section contains the balance recovery response to perturbation.

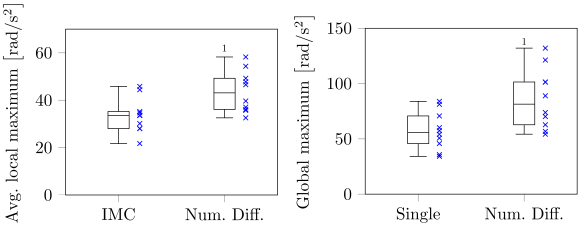

Fig. S5 Values of the personalized threshold extracted from the overground walking baseline measurements. The box plots show the threshold for the averaged local maximum and the threshold for the global maximum of all participants. The respective values across all subjects are displayed with crosses. In each plot the lower whisker corresponds to the smallest value, the upper whisker to the largest value and the box is formed by the median and the 25 % and 75 % quartiles.

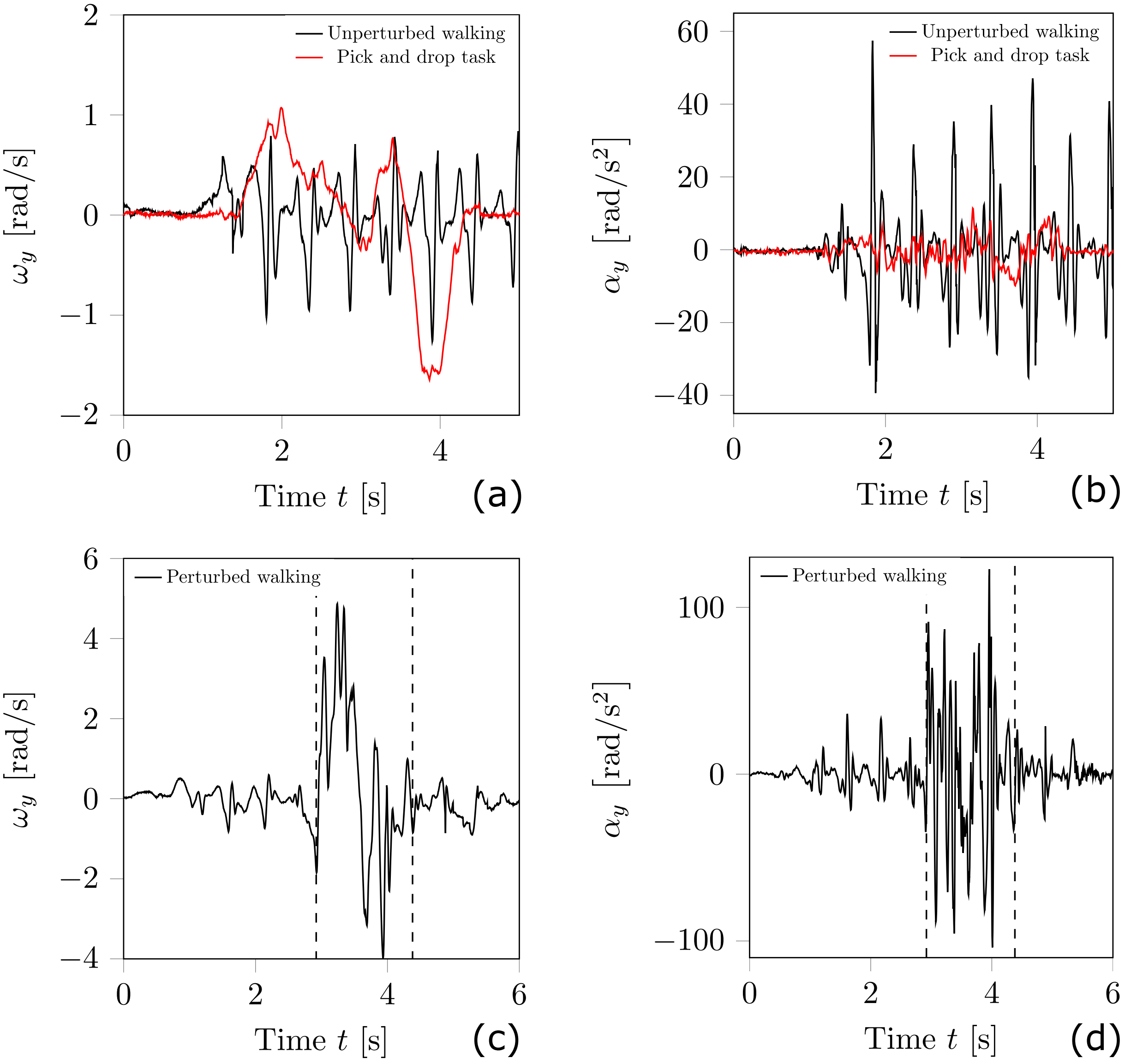

Fig. S6 Comparison of trunk kinematics for picking and dropping a box, walking unperturbed as well as perturbed. The trunk angular velocity and angular acceleration vector coordinate of the transverse axis (principle y-axis of the trunk) of one participant picking and dropping a 5 kg box from an initially upright standing position and walking unperturbed (a, b) as well as perturbed (c, d) at a given velocity (1.4 m/s) are shown.
