# Supplementary material for: A wearable sensor and framework for accurate remote monitoring of human motion
Source: Commun Eng. 2024 Jan 30;3:20. doi: 10.1038/s44172-024-00168-6 (PMC11101649; doi:10.1038/s44172-024-00168-6)
Supplement: Supplementary file 2 — Reporting Summary [file 44172_2024_168_MOESM2_ESM.pdf]

nature portfolio

Corresponding author(s): Maximilian Gießler

Last updated by author(s): Dec 26, 2023

Reporting Summary

Nature Portfolio wishes to improve the reproducibility of the work that we publish. This form provides structure for consistency and transparency in reporting. For further information on Nature Portfolio policies, see our Editorial Policies and the Editorial Policy Checklist.

Statistics

For all statistical analyses, confirm that the following items are present in the figure legend, table legend, main text, or Methods section.

n/a	Confirmed	
☐	☒	The exact sample size (*n*) for each experimental group/condition, given as a discrete number and unit of measurement
☐	☒	A statement on whether measurements were taken from distinct samples or whether the same sample was measured repeatedly
☐	☒	The statistical test(s) used AND whether they are one- or two-sided<br>*Only common tests should be described solely by name; describe more complex techniques in the Methods section.*
☒	☐	A description of all covariates tested
☐	☒	A description of any assumptions or corrections, such as tests of normality and adjustment for multiple comparisons
☐	☒	A full description of the statistical parameters including central tendency (e.g. means) or other basic estimates (e.g. regression coefficient) AND variation (e.g. standard deviation) or associated estimates of uncertainty (e.g. confidence intervals)
☐	☒	For null hypothesis testing, the test statistic (e.g. *F*, *t*, *r*) with confidence intervals, effect sizes, degrees of freedom and *P* value noted<br>*Give P values as exact values whenever suitable.*
☒	☐	For Bayesian analysis, information on the choice of priors and Markov chain Monte Carlo settings
☒	☐	For hierarchical and complex designs, identification of the appropriate level for tests and full reporting of outcomes
☐	☒	Estimates of effect sizes (e.g. Cohen's *d*, Pearson's *r*), indicating how they were calculated

Our web collection on statistics for biologists contains articles on many of the points above.

Software and code

Policy information about availability of computer code

Data collection	Regarding the real-world application, we used a custom code developed in C++. The introduced sensor is operating with a custom code and uses the framework "Robot operating system 2 - Galactic" to publish the data in a network. Regarding the simulated data collection, we used the open-source simulator "Webots 2022b" and custom code written in C++.
Data analysis	All analyses were performed using SPSS Statistics (v27, IBM; Chicago, IL, USA) and MATLAB (2020b, MathWorks, Natick, MA, USA).

For manuscripts utilizing custom algorithms or software that are central to the research but not yet described in published literature, software must be made available to editors and reviewers. We strongly encourage code deposition in a community repository (e.g. GitHub). See the Nature Portfolio guidelines for submitting code & software for further information.

Data

Policy information about availability of data

All manuscripts must include a data availability statement. This statement should provide the following information, where applicable:

- Accession codes, unique identifiers, or web links for publicly available datasets
- A description of any restrictions on data availability
- For clinical datasets or third party data, please ensure that the statement adheres to our policy

All datasets generated during and/or analysed during the current study are available on Figshare with identifier https://doi.org/10.6084/m9.figshare.24781506.v1

Human research participants

Policy information about studies involving human research participants and Sex and Gender in Research.

Reporting on sex and gender	The current study did not aim to investigate potential effects of either sex or gender on balance recovery as it was to demonstrate in general whether our technology can assess human motion. Thus, no respective analyses were performed. Since we inspected statistical assumption for each variable of interest (often including pooled data of 9 male and 2 female participants; self-reported prior to participation; as noted in the in the methods section of the version of our manuscript submitted for review), we are confident that we have accounted for any potential statistical bias caused by sex or gender.
Population characteristics	As described in the in the methods section of our manuscript submitted, our participants (data as averages and standard deviations 29 ± 6 years of age, 1.76 m ± 0.12 m body height, 77.8 kg ± 8.8 kg body mass) were healthy (i.e. neurological or musculoskeletal injuries or impairments limiting locomotion) and moderately active.
Recruitment	Participants were recruited based on interest received upon information given to undergraduate and postgraduate students and staff of London South Bank university. We are confident that there were no bias caused by the recruitment procedure.
Ethics oversight	London South Bank University; School of Applied Science.

Note that full information on the approval of the study protocol must also be provided in the manuscript.

Field-specific reporting

Please select the one below that is the best fit for your research. If you are not sure, read the appropriate sections before making your selection.

☒ Life sciences ☐ Behavioural & social sciences ☐ Ecological, evolutionary & environmental sciences

For a reference copy of the document with all sections, see nature.com/documents/nr-reporting-summary-flat.pdf

Life sciences study design

All studies must disclose on these points even when the disclosure is negative.

Sample size	The current sample size was chosen based on our previous studies and respective effect sizes (e.g. Werth et al. 2022, Sci Rep 12) related to adaptation phenomena on balance control due to repeated perturbations; e.g. partial eta squared with n = 10 ranging from 0.536 to 0.775, depending on the variable of stability performance analysed).
Data exclusions	For 'overground gait perturbations', testing of two participants could not be completed due to technical issues. Furthermore, due to safety harness interference and various signal artefacts not all of the 72 perturbation trials for the remaining nine participants could be analysed, i.e sometimes only one trial was included to represent early (trial 1 or 2), mid (trial 4 or 5) or late (trial 7 and 8) phases. As for the 'overground gait perturbation' task, testing of one subject could not be completed due to technical issues during the 'lean-and-release' task.
Replication	For the tasks 'repeated overground-based/treadmill-based gait trip-like perturbations' we expected trial-by-trial adaptation phenomena hence did not perform any reproducibility assessment. However, we previously performed several investigations on all three balance tasks and revealed consistent effects across studies whilst using different population groups and study designs. Thus, we are confident that all three tasks are reproducible.
Randomization	All participants underwent the same experimental protocol (i.e. only one experimental group) hence randomization was not relevant for the current study.
Blinding	We only assessed one group hence blinding was not relevant for the current study.

Reporting for specific materials, systems and methods

We require information from authors about some types of materials, experimental systems and methods used in many studies. Here, indicate whether each material, system or method listed is relevant to your study. If you are not sure if a list item applies to your research, read the appropriate section before selecting a response.

Materials & experimental systems

n/a	Involved in the study
☒	☐ Antibodies
☒	☐ Eukaryotic cell lines
☒	☐ Palaeontology and archaeology
☒	☐ Animals and other organisms
☒	☐ Clinical data
☒	☐ Dual use research of concern

Methods

n/a	Involved in the study
☒	☐ ChIP-seq
☒	☐ Flow cytometry
☒	☐ MRI-based neuroimaging
